# Physical Performance, Body Composition, and Oral Health in Community-Residing Older Adults: A Cross-Sectional Study

**DOI:** 10.3390/geriatrics9040089

**Published:** 2024-06-26

**Authors:** Maria Esther Irigoyen-Camacho, Maria Consuelo Velazquez-Alva, Marco Antonio Zepeda-Zepeda, Irina Lazarevich, Antonio Castano-Seiquer, Javier Flores-Fraile

**Affiliations:** 1 Health Care Department, Metropolitan Autonomous University, Calzada del Hueso 1100, Col. Villa Quietud, Mexico City 04960, Mexico; mzepeda@correo.xoc.uam.mx (M.A.Z.-Z.); iboris@correo.xoc.uam.mx (I.L.); 2 School of Dentistry, University of Seville, C. Avicena, s/n, 41009 Seville, Spain; 3 Department of Surgery, Faculty of Medicine, University of Salamanca, C. Alfonso X el Sabio, s.n., 37008 Salamanca, Spain; j.flores@usal.es

**Keywords:** older adults, physical performance, percent body fat, nutritional status, oral health-related quality of life, type 2 diabetes mellitus

## Abstract

Physical activity is essential for healthy aging. This study aimed to identify an association between physical performance, body fat percentage (%BF), and the perception of oral health-related quality of life (OHRQoL) in independent older adults. Method: A group of active older adults was selected from a government-sponsored reunion center in Mexico City. OHRQoL was assessed using the General Oral Health Index (GOHAI), and nutritional status was assessed using the Mini Nutritional Assessment (MNA) tool. A short physical performance battery (SPPB) was applied, and, for body composition, DXA (dual X-ray absorptiometry) was conducted. Data were analyzed using logistic regression models, and marginal probabilities were obtained. Results: This study involved 366 participants; their mean age was 73.9 (±6.2) years, and 24.9% had type 2 diabetes mellitus (T2DM). OHRQoL information revealed that pain or discomfort in the oral cavity was perceived by 63.9% of the older adults during the previous three months. The SPPB score was low in 159 (43.44%) participants. The logistic regression model revealed that age (OR = 1.13, *p* < 0.001), T2DM (OR = 2.10, *p* = 0.009), the risk of malnutrition/malnutrition (OR = 1.76, *p* = 0.047), high %BF (OR = 1.09, <0.001), and poor OHRQoL (OR = 1.96, *p* = 0.009) were associated with deteriorated physical performance. Conclusion: OHRQoL self-perception, excess body fat, and nutritional status impacted physical performance. Aging well requires a comprehensive approach.

## 1. Introduction

Population aging presents a significant challenge to the healthcare system. In 2022, there were 771 million older people worldwide; this is expected to rise to 994 million by 2030 and to 1.6 billion by 2050, representing 16% of the global population [1]. Mexico’s demographic structure is rapidly changing. Currently, there are 15.1 million people over the age of 60 (12.1% of the population) [2], and this is expected to reach 33.3 million by 2050 (28%) [3].

Physical performance can be defined as the ability to integrate physiological mechanisms into coordinated movements to perform physical functions, such as standing up, sitting in a chair, walking, or climbing stairs [4]. Aging is frequently accompanied by a decrease in physical performance and functional capacity [5,6]. The short physical performance battery (SPPB) is a tool widely used in older age groups to assess physical performance [7]. The SPPB consists of three subtests: the chair rise test, the balance test, and the gait speed test. A literature review concluded that a low SPPB score was associated with greater all-cause mortality, while a high score reflected greater survival probability [8]. Evidence suggests that the SPPB may predict disabilities, and it is associated with nutritional status, hospitalizations, frailty, the chewing index, and multimorbidity and mortality in the elderly [9,10,11,12,13].

It is well established that body fat percentage (%BF) increases with age [14], and, worldwide, the prevalence of obesity is increasing rapidly [15,16]. In Mexico, obesity is estimated to be 40% and 26% in female and male older adults, respectively [17]. An association between obesity and mortality has been demonstrated in different studies [18,19,20]. Furthermore, %BF is frequently associated with comorbidities (cardiovascular diseases, arterial hypertension, diabetic mellitus type 2, metabolic syndrome, etc.) and disability [15].

During the aging process, changes in body composition, including progressive muscle loss, affect physical performance [21]. Body composition studies have found that high excess body fat is associated with poor physical performance [22], particularly in women [23]. Notably, trunk fat mass has been linked to poor balance and deteriorated physical performance in both men and women [24]. Additionally, physical performance plays an important role in determining longevity and quality of life.

The self-perception of quality of life has been linked to physical performance and nutritional status in older adults [25]. The Costa Rican Longevity and Healthy Aging Study found that oral health status impacted general health perception. In particular, an association between severe tooth loss and poor self-rated health was found. Moreover, oral health was the fifth most important condition affecting the perception of general health in this representative sample of Costa Rican older adults [26]. The literature on the impact of oral health on physical performance is limited, and some findings are inconsistent [27,28,29]. The Swedish National Study on Aging and Care in Kungsholmen identified an association between the loss of a large number of teeth and slow walking speed but no association with eating difficulties [30]. In a cross-sectional study of Japanese adults, in which the relationship between oral health and muscle mass and functioning was examined, distinct associations were observed by sex. For women, fewer teeth were linked to slower performance on the timed 10-m walk test, an association not found in men. Conversely, in men, a lower skeletal muscle mass, as estimated using a bioelectrical impedance analysis, was associated with tooth loss, an association not observed in women. This suggests that different factors may be at play for men and women regarding tooth loss and muscle mass [31].

In cohort studies of older adults in the UK and USA, it was observed that those with difficulties in chewing and deteriorated dentition were at an increased risk of a slower chair stand speed than those without dental problems in the British group. In the USA group, the perception of poor oral health and edentulism were associated with more time spent on a 20 m regular walk [32]. A follow-up study in Japan found that the number of natural teeth in contact during mastication was associated with leg extension power, a measure of lower limb strength during movement. Furthermore, the time being able to stand on one foot was associated with dental occlusion [33]. These findings highlight the importance of maintaining a functional occlusion during aging.

Previous studies have investigated some aspects of oral health self-perception, such as eating difficulties or the general perception of oral health in older adults [30,33]. However, a more comprehensive evaluation of the perception of oral health-related quality of life could be valuable in elucidating the association between oral health self-perception and physical performance. The ability to perform normal oral functions, such as eating, swallowing, and smiling, impacts the self-assessment of the oral health-related quality of life (OHRQoL) [34]. OHRQoL is a multidimensional construct that reflects an individual’s subjective perception of the impact of their oral health on their overall well-being and ability to perform daily activities [35]. It is influenced by personal and social expectations and experiences, and it is closely related to oral health conditions [34].

An analysis of the relationship between body composition and physical performance, as well as information on the self-perception of oral health-related quality of life (OHRQoL), may provide additional insights into the role of different aspects of health in affecting physical performance in older adults. In this context, this investigation aimed to identify the association between %BF, nutritional status, OHRQoL, and physical performance among independent older adults.

## 2. Materials and Methods

### 2.1. Design and Participants

This study used a cross-sectional design. Participants were older adult women and men who attended a public community center in the south of Mexico City. The area’s population was approximately 15,250 residents in 2020. According to socioeconomic indicators, it is classified as a middle-income neighborhood [36]. It is estimated that 27.6% of the population in this community is 60 years old or older. The female population accounts for 57.7%, and the male population accounts for 42.3% [37]. About 850 older adults were registered at the selected center. This center offers free access to people 60 or older and receives support from Mexico City’s government. Attendees can participate in dance, knitting, painting, English, and singing lessons. In addition, there is a gym offering spinning, yoga, and Tai Chi classes. A swimming pool, soccer field, and basketball court are also available. Among women, dancing, physical fitness training, gymnastics, aerobic exercise, swimming, and Tai Chi were the most popular activities. Most men prefer soccer, tennis, or racquetball. Some train in the gym and play squash, while others practice basketball. Usually, attendees practice physical activities two or three times a week.

To invite and recruit study participants, the researchers used the following procedure: First, they sent an official letter to the Social and Sports Center for Older Adults requesting permission to conduct the study. Detailed information about the assessment (free of charge), as well as the dates and times of the visit, was included in this letter. Second, after obtaining permission to conduct the study, the researchers placed posters outside the sports center entrances inviting people to participate in the study. The researchers provided older adults with comprehensive and detailed information regarding the evaluation process and how long it would take. The researchers also approached individuals and asked them if they would be interested in undergoing an evaluation of their physical performance and nutrition status, as well as completing a questionnaire related to their general health and, specifically, their oral health.

After obtaining informed consent, the evaluation was conducted in two stages. The first stage was carried out at the sports center (which could be completed on the same day, as the process took approximately 45–60 min). In the second stage, participants were required to attend the university campus at a mutually agreed upon time and date, approximately a 20-min walk away. Eligibility was determined through a screening phase. Information on medical history was collected to assert health status and medication use, and the Short Portable Mental Status Questionnaire was applied to evaluate cognitive impairment, both of which were performed by an experienced examiner.

To calculate the sample size, odds ratios (ORs) were used as a measure of association between the major variables, with an expected OR of 2, considering the relationship between oral health status and physical performance [38]. Additionally, for sample size calculations, we assumed a Type I error of 0.05, with a power of 0.80, and a prevalence of 0.30 in the absence group (low physical performance in the group with a favorable OHRQoL) [39]. A total of 348 individuals were estimated to be recruited for the study. The data of 366 participants were analyzed.

### 2.2. Inclusion Criteria

An examination was offered to men and women 60 years or older who were registered at the selected community center. The study was open to individuals with chronic diseases such as hypertension and type 2 diabetes mellitus (T2DM) if they were under medical control. Participants were also required to complete questionnaires and undergo nutritional and body composition assessments using dual X-ray absorptiometry (DXA). Signing a consent form before participating in the study was also necessary.

### 2.3. Exclusion Criteria

Among the exclusion criteria were severe medical conditions, recent hospitalizations, cancer treatment within the previous six months, edema, and recent bone fractures. These conditions are associated with fatigue, anorexia, and sarcopenia, and they may negatively affect body composition and severely limit physical performance [40,41]. Thus, individuals with these conditions were excluded to avoid confounding [42]. Additionally, individuals with physical disabilities requiring wheelchairs were excluded because they could not perform the physical tests, which evaluated the patients’ ability to maintain three standing positions and their speed of walking and getting up from a chair. Furthermore, individuals with severe cognitive impairment were excluded from the study. This was because they were required to recall information and answer questions regarding their dietary habits and oral health [43].

**Figure 1:**
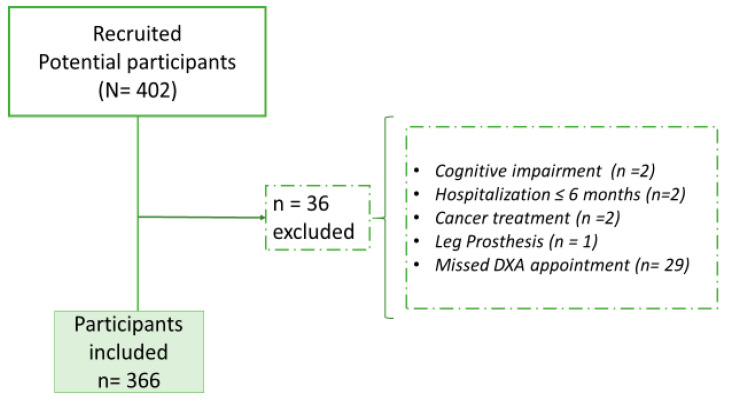
A flowchart illustrating the process of selecting study participants. The right rectangle displays the reasons for excluding 29 individuals who did not meet the study eligibility criteria.

The data analysis did not include information regarding the older adults who failed to attend their scheduled DXA appointments. About 464 attendees were approached, and 402 were interested in participating in the study. Figure 1 shows a flowchart of the participants. In total, 402 older adults were recruited; 7 were excluded due to health-related exclusion criteria, and 29 failed to attend the DXA examination appointment. There was no significant difference in the body mass index (BMI) between those who did not attend their appointment and those who did (*p* = 0.720).

### 2.4. Ethics

This study was conducted in accordance with the Declaration of Helsinki ethical standards for medical research involving human subjects. Recruitment and data collection were conducted from November 2023 to February 2024. The research protocol was approved by the Council of the Division of Biological and Health Sciences and by the Ethics Committee of the Universidad Autonoma Metropolitana-Xochimilco (approval number: DCBS.CD.52.17).

### 2.5. Anthropometry

A nutritionist certified in kinanthropometry (ISAK International Certification in Kinanthropometry) performed weight and height measurements in accordance with recommended procedures [44]. A portable electronic digital scale with an integrated stadiometer was used to measure body weight and height. Waist circumference was measured using a fiberglass measuring tape placed between the lower rib and the iliac crest, with the results expressed in centimeters. Body mass index (BMI) was calculated by dividing the body weight (kilograms) by the square of the height (meters). This index is widely used to assess overweight and obesity. An individual with a high BMI is more likely to suffer from decreased functional capacity and experience problems with gait speed and balance [45]. The World Health Organization (WHO) classification system for BMI was applied. The Lipschitz BMI criteria were also reported for classifying excess weight (BMI over 27 kg/m^2^) [46].

### 2.6. Nutritional Assessment

The Mini Nutritional Assessment (MNA) tool was applied to assess nutritional status. This is a widely used tool to evaluate nutritional status in older adults, and both malnutrition and malnutrition risk can be identified [47]. The MNA contains 18 items, with a score range of = to 30; a score ≥24 was considered well nourished, and a score of 17 or below was identified as malnourished [48]. Anthropometric measurements in the MNA include weight, height, and circumferences of the calf and upper arm. A nutritionist assessed these measurements following standardized procedures (International Society for the Advancement of K anthropometry, ISAK).

### 2.7. Physical Performance

The short physical performance battery (SPPB) measures older adults’ functional capacity and physical ability. The SPPB method is considered an adequate, relatively simple tool for evaluating physical function in various settings [49]. Three tests are included in the battery: balance, gait speed, and chair rise tests. The SPPB measures physical ability on a scale of 0 to 12. Low scores indicate poor performance [49]. A score of 8 points or less suggests a decline in physical performance [14].

### 2.8. Body Composition Assessment

Dual X-ray absorptiometry (DXA) was applied to evaluate body composition. This technology is considered adequate to evaluate body composition and requires low radiation exposure [50]. Participants in the DXA test were required to wear light clothing without zippers or metal decorations. To prevent interference with the measurements, all jewelry, coins, and keys were required to be removed. A whole-body scan was conducted using Hologic Discovery QDR Series DXA equipment in accordance with the manufacturer’s instructions. The procedure was carried out by a trained laboratory technician. Before each session, the equipment was calibrated with the manufacturer’s phantom. Each participant was instructed to lie face up on the platform table while aligning their body with the midline of the table and keeping their toes together during the scanning procedure. The participants were also asked to maintain a still position during the body scan.

### 2.9. Oral Health-Related Quality of Life Assessment

Oral health-related quality of life (OHRQoL) was assessed using the General Oral Health Index (GOHAI) [51]. There is more information available about this questionnaire’s psychometric properties than about other self-administered questionnaires, and it is widely used among older adults [52]. The validated Mexican GOHAI version was applied [53]. This questionnaire has 12 items divided into physical, psychosocial, and pain/discomfort components. The physical component refers to oral cavity functions, including masticatory capacity, swallowing, and talking. The psychological component involves an individual’s appearance, smiling, and avoidance of social events. The pain and discomfort component includes oral symptoms and medication used to control pain. The GOHAI questionnaire includes items regarding the conditions present over the previous three months. The answers are rated on a Likert scale from 1 to 5. The scale varies from 12 to 60. Higher scores indicate better oral health perceptions.

### 2.10. Statistical Analysis

The data are described using means and proportions. The difference between means was determined using nonparametric techniques (Mann–Whitney U-test), considering the variables’ non-normal distribution. Using the χ^2^ test, associations between categories were analyzed. Multiple logistic regression models were conducted for the SPPB score (cut-off = 8). In the multiple regression model constructed for physical performance as a dependent variable, interactions between nutritional status, body fat percentage, and oral health-related quality of life were examined. Independent variables included in the multiple regression model were those with a *p* < 0.20 in the bivariate analysis or those theoretically necessary. The goodness of fit of the logistic regression models was tested using the Hosmer–Lemeshow statistic. Collinearity between independent variables in the model was analyzed using the Variance Inflation Factor (VIF). The literature suggests a value VIF > 10 as an indicator of multicollinearity [54]. In addition, marginal probabilities were estimated and are presented graphically. The significance level of the hypothesis test was set to *p* < 0.05. STATA V15 was used for a statistical analysis.

## 3. Results

The total number of participants was 366, of whom 74.6% (n = 273) were women, and 25.4% (n = 93) were men. The mean age of the study group was 73.9 (±6.2), and no significant difference in age was found between women and men (*p* = 0.322). Among the participants, the mean BMI was 27.3 kg/m^2^ (±4.2). Lipschitz’s criteria showed that 48.4% of the participants had excess weight. Table 1 presents the sociodemographic characteristics of the participants by sex. About half (45.1%) of the participants were married or lived with a partner, and one-third were widows. It was more common for men to be married and for fewer of them to be widowed than for women (*p* < 0.001). One-quarter of the participants (24.9%) had T2DM, and more than half (54.9%) had hypertension. The prevalence of T2DM (*p* = 0.424) and HT (*p* = 0.051) was not significantly different between women and men. According to the MNA results, more women than men were at risk of malnutrition (*p* = 0.002). Women had a higher BF% and a lower fat-free mass (kg) (FFM) (*p* < 0.001) than men. The OHRQoL evaluation was similar for women and men. According to the SPPB score, more women had limitations in physical performance than men (*p* = 0.023). Regarding the GOHAI components, experiencing physical difficulties during eating, swallowing, or talking was perceived by 55.7%; regarding the psychosocial component, 61.20% were impacted; and pain or discomfort in the oral cavity was perceived by 63.9% of the older adults during the previous three months.

The SPPB score was low (SPPS ≤ 8) in 159 participants (43.4%) and high in 207 participants (56.6%). The SPPB results were lower in older participants, indicating a decrease in capabilities with age. A mean difference of approximately three years was observed (3.45 IC95% (2–20, 4.67) (*p* < 0.001) by SPPB score (Table 2). A higher prevalence of hypertension (*p* = 0.004) and DMT2 (*p* = 0.005) was found in the older adults with impaired physical performance. A higher percentage of participants at risk of malnutrition or malnourishment had lower physical performance scores than those with a normal nutritional status (*p* = 0.002). Additionally, a lower FFM (*p* < 0.001) and higher BF% (*p* < 0.001) were identified in the group with poor physical performance. Furthermore, a greater impact on the OHRQoL total score and on each of the dimensions (physical (*p* < 0.001), psychosocial (*p* = 0.007), and oral pain/discomfort (*p* = 0.006)) was found in the group with lower physical performance (Table 2).

**Figure 2:**
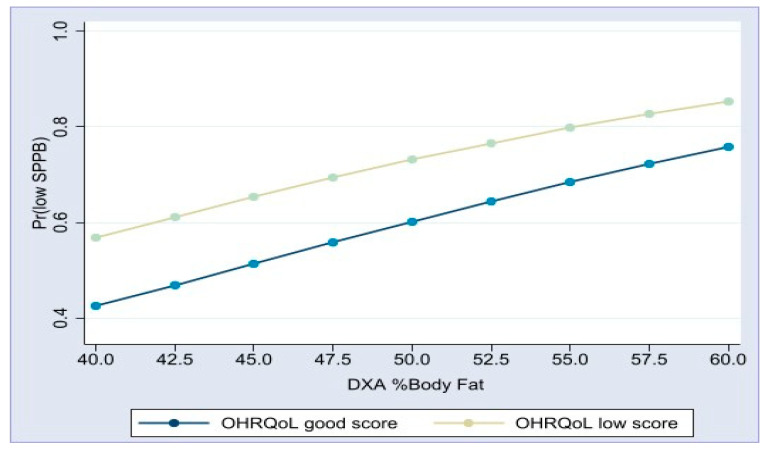
The graph shows that, as body fat percentage (%BF) increases, the likelihood of having low physical scores increases. The two-line diagram shows the probability of poor physical performance in two groups. The line with higher probability corresponds to the group with poor scores on the General Oral Health Assessment (GOHAI), and the line with lower probabilities corresponds to the group with better scores on the GOHAI questionnaire.

Table 3 presents the results of the logistic regression models for physical performance (SPPB ≤ 8). In the bivariate models, age (*p* < 0.001), sex (0.024), hypertension (0.041), DMT2 (*p* < 0.001), nutritional status (*p* = 0.005), BF% (*p* < 0.001), and OHRQoL (*p* < 0.001) were statistically significant. In the multivariate model, age (*p* < 0.001) was associated with poor SPPB results, and hypertension was not (*p* = 0.740). Additionally, poor nutritional status (*p* = 0.047), higher BF% (*p* < 0.001), and T2DM (*p* = 0.009) were associated with lower physical performance. Furthermore, OHRQoL (GOHAI score) was associated with the SPPB (*p* = 0.009). Interactions between nutritional status, body fat percentage, and OHRQoL were not statistically significant in the model (*p* > 0.160). Figure 2 depicts the predicted probability of having a low SPPB score and the BF% by the GOHAI score. It was observed that, as the %BF increased, the probability of low physical scores increased. Two lines are presented: the line with higher probabilities corresponds to the group with poor GOHAI scores, and the group with better GOHAI scores presents a lower likelihood of impaired physical performance. For example, for 40%BF and 45%BF, the probability of having low physical scores in the group with high GOHAI scores was 0.43 and 0.52, respectively. The probabilities were higher in the group with poor GOHAI scores, at 0.57 and 0.65, respectively.

## 4. Discussion

This study found a significant association between oral health-related quality of life and physical performance, even after adjusting for key covariates. Notably, the perception of OHRQoL was significantly associated with objective physical performance measures. Accordingly, in a prospective cohort study that included older adults from the UK and USA, the self-perception of oral health was associated with gait speed [32]. Previous research has analyzed the relationship between dental status and physical performance measures. For instance, investigations in China, Japan, and Sweden found that tooth loss or chewing difficulties was associated with muscle strength, bone mineral content, and the self-perception of general health [28,30,55]. Studies conducted in England and Japan among very old individuals (85 years or older) revealed that difficulties in eating, edentulism, and dry mouth were associated with weak hand grip strength and slow gait speed [56]. Thus, addressing oral health problems in older adults could potentially benefit their physical functioning. This approach, however, may not be feasible in many settings, including in Mexico, due to dental rehabilitation costs.

The present study utilized an instrument (GOHAI) comprising three components: pain/discomfort, physical, and psychosocial components. More than half of the participants were affected by the physical and psychosocial components, and more than two-thirds experienced pain or discomfort associated with their mouth. Using self-report instruments to assess OHRQoL has the advantage of avoiding a direct oral examination, which could be challenging in certain environments.

Several mechanisms may underlie the relationship between oral health and physical performance. One mechanism may be related to masticatory deficiency and poor diet; this may lead to poor muscle strength, which may further impact physical performance. An analysis of the US National Health and Nutrition Examination Survey (NHANES) 2001/2012, including adults 50 years or older, revealed an association between tooth loss, denture use, and frailty. Nutritional status explained 31% of the associations between oral conditions and musculoskeletal frailty [27].

Another possible mechanism is related to chronic inflammation due to oral infections [57,58]. A follow-up study in Sweden identified an association between tooth loss and slow walking speed. When inflammation was included in the model (assessed via C-reactive protein), the association between tooth loss and slow walking speed was no longer statistically significant. It was suggested that chronic inflammation may be involved in the relationship between walking speed and tooth loss over time [30]. However, a study in Korean older adults did not find a significant association between periodontal diseases and osteoporotic fracture risk. The authors indicated that the nature of the index used to evaluate periodontitis, which is affected by the number of teeth present, may have contributed to the lack of association [59].

The relationship between body balance and tooth loss is another potential mechanism. Masticatory muscles and the periodontal ligament have proprioceptive receptors that may contribute to body balance [60]. Tooth loss results in a reduction in periodontal receptors, which decreases nerve center stimulation. A study using magnetic resonance imaging to identify cortical activation in distal muscles found a correlation between tooth clenching and the activation of remote muscles during a handgrip test [61]. Additional research is necessary to determine the relationship between periodontal membrane receptors, the cortical response, and physical performance. In Japanese older adults, one-leg standing time was related to the number of occluded natural teeth [33]. Mastication forces exerted on the teeth are transmitted to distal members of the body; this occurs through the propagation of forces via the musculoskeletal system [62]. Additionally, the trigeminal nerve responds to occlusal forces and oversees the motor control of the mastication muscles. This V cranial nerve plays a vital role in the response to occlusal forces, affecting physical performance by regulating proprioception, motor control, pain modulation, and integration with other systems, such as the oculomotor and vestibular systems, to coordinate movements and maintain posture [63]. Maintaining adequate dental occlusal contact may benefit balance in older adults.

In the current study, T2DM participants were more likely to experience poor physical performance. Similarly, a study conducted on Peruvian older adults found that diabetic patients were more likely to encounter gait difficulties and balance problems than non-T2DM patients [64]. Older adults may experience both T2DM and physical disability, which are associated with an increased risk of falls and other geriatric syndromes [65]. A bidirectional relationship has been suggested between physical disability and T2DM. Diabetic neuropathy patients, for example, may be less physically active, which contributes to greater disability [66]. T2DM patients have a significantly higher rate of physical decline than people without T2DM [67]. Exercise programs that prevent muscle loss are essential, particularly in countries with a high T2DM prevalence.

The results of the present study revealed a higher probability of poor functional performance with increasing body fat percentage. Body composition has been associated with physical performance in older adults from different countries. In a study with a longitudinal design in patients attending a clinic in California, USA, an increase in fat mass percentage and a decrease in fat-free mass were associated with greater functional disability [68]. A high fat mass index also predicted a poorer health-related quality of life in a group of Finnish adults followed for 10 years [69]. Among octogenarians in Brazil, a higher appendicular skeletal muscle mass was positively related to improved physical performance, and a negative association was also found between the SPPB and body fat percentage [22]. It is notable that, despite the older age of the participants, good body composition accompanied good physical performance. A cross-sectional study of a representative sample of Finnish adults found that the association between body fat percentage and maximum walking speed was the strongest between the ages of 60 to 69 and 70 to 79. This explained between 21% and 17% of the variation in walking speed, respectively; however, this association decreased in older age groups [70]. The effects of aging on body composition and physical performance require further investigation.

Body fat and physical performance may be connected to a higher risk of chronic diseases such as cardiovascular disease, high blood pressure, and certain cancer types. In turn, these clinical conditions can severely limit physical performance and independence in older people [71,72]. Additionally, excess body fat can cause additional stress on the joints and muscles. This may lead to an increased risk of falls and injuries and hamper everyday activities such as walking, climbing stairs, or getting up from a chair [73]. Moreover, excess fat mass may also affect energy metabolism, resulting in a faster rate of fatigue. Intra-abdominal fat may increase exercise intolerance [74]. Visceral adipose tissue produces pro-inflammatory cytokines that contribute to the development of chronic inflammation, poor muscle quality, and poor performance during exercise [75]. A proactive approach to preventing obesity and promoting a more active lifestyle among older adults is essential in health programs for older people.

In this study, the participants at risk of malnutrition/malnourishment were more likely to have poor physical performance. In Spanish, Peruvian, and Norwegian older adults, higher SPPB results were observed in groups with a better nutritional status [13,76,77]. Further, a longitudinal study involving community-dwelling older adults in the UK found that a higher SPPB score at baseline was protective against deterioration in nutritional status [78]. It has been reported that malnutrition is only associated with measures of dynamic physical performance, such as gait speed and the time it takes to get out of a chair, and not with static physical performance. It is argued that physical performance measures have individual value concerning nutritional status. Therefore, it is worth considering both dynamic physical performance and static physical performance in the relationship with nutritional status. Assessing nutritional status over a longer period is also critical to understanding the impact on specific areas of physical performance [79].

Physical performance and nutritional status are crucial factors influencing older adults’ health and quality of life. Nutrition plays a significant role in managing and preventing chronic diseases, as well as in enhancing the immune response [80]. An adequate calorie and nutritional intake is necessary to maintain optimal energy and resistance levels [81]. Healthy diet patterns, such as the Mediterranean diet, are crucial to prevent body adiposity. A study in community-dwelling Italian older adults found that those who follow a healthy lifestyle, including a Mediterranean-style diet, have a lower body fat index [82]. This diet has a high proportion of dietary fiber, antioxidants, and healthy fats, which contributes to better weight management and decreased fat accumulation. Furthermore, the diet’s anti-inflammatory properties and positive effects on metabolism support overall health, making it an effective strategy for decreasing obesity, reducing the risk of sarcopenia, and promoting longevity [83,84].

An integrated approach that includes resistance exercise and a diet rich in protein, essential amino acids, and vitamin D is recognized as effective in promoting muscle growth and improving functional capacity in this age group [85]. According to current recommendations, older adults should consume between 1 and 1.2 gr of protein per kilogram of body weight per day. This may help in maintaining muscle mass; gaining strength; and improving physical performance, independence, and quality of life [86]. The early detection of malnutrition or the risk of malnutrition in older adults is critical to prevent the deterioration of physical performance.

Limitations and Strengths: This study has some limitations. First, its cross-sectional design prevents the determination of causal relationships between physical performance, body composition, and oral health among older adults. Longitudinal studies are needed to better understand these associations’ directionalities and possible causality mechanisms. Second, the results may be susceptible to biases. Recall and social desirability biases may be associated with self-reported data on some aspects of nutritional status included in the MNA. However, anthropometric data, an essential part of a nutritional evaluation, were meticulously recorded by a certified nutritionist, strengthening the validity of the results. The exclusion criteria could have led to selection bias; however, they are required to avoid confounding factors, creating a more homogeneous study group and allowing for the objective of the study to be achieved [42].

We excluded patients who experienced recent hospitalizations, fractures, and edema; who were undergoing cancer treatment; and who were severely ill. This is because these conditions strongly impact body composition and physical performance. Seven individuals (1.7% of potential participants) fitted into these categories. Additionally, data from several participants (n = 29) were excluded because they failed to attend their DXA appointments. To determine whether body weight influenced attendance, the body mass index of those who completed the DXA assessment and those who did not was compared. No significant difference in BMI was found between these groups, thereby reducing the probability of selection bias.

Third, the generalizability of the results is limited. The participants in this study were recruited from a social sports center in a middle-income neighborhood in Mexico City, and they may not be representative of older adults from low-income neighborhoods. Considering this, it may not be appropriate to extrapolate the results to these populations.

However, the study population included participants with chronic conditions such as DM2 and HT. Mexican older adults have a high prevalence of both DMT2 and HT, at 37.0% and 74.8%, respectively, among those 60 years or older [87,88]. It was necessary to include these groups to improve the external validity of the results [87]. The study group had a similar prevalence of these conditions to a large cohort study of older adults living in Mexico City [89]. Considering this, we believe that our findings are relevant to urban populations with similar epidemiological characteristics.

Self-reported oral health perceptions can provide valuable insights, particularly in populations where oral examinations may prove difficult. This study has several strengths, including a comprehensive evaluation of oral health perception using the GOHAI and the use of the DXA method. This is considered an adequate method for body composition assessment, is quick, and produces low-radiation exposure [50]. The response rate in our study was high, which contributes to the internal validity of the results. Although this study has some limitations, it provides valuable insights into the relationship between physical performance, body composition, and oral health in older independent adults living in urban settings.

Health-related issues among older adults in urban regions of large cities should be studied to understand their specific health conditions, mainly exacerbated by inadequate lifestyles. As a result of geriatric research, it may be possible to develop better community programs, support networks, and public policies that promote healthy aging. Specifically, tailored health programs can be implemented to meet the needs of this population. Healthy older adults will be able to contribute more actively to society for a longer period [90].

## 5. Conclusions

The results of the present study emphasize the impact of self-perceived oral health, body fat percentage, and nutritional status on physical performance among urban community-dwelling older adults. The results show that self-perceived oral health significantly influences physical performance. Additionally, body fat percentage and nutritional status influence physical performance. It is essential to consider the interrelationship between healthy nutrition and adequate body composition, particularly for older adults. Furthermore, oral health is a crucial component of general health and is associated with physical performance.

## Figures and Tables

**Table 1:** Characteristics of community-dwelling participants by sex.

Characteristics	Women (74.6%)	Men (25.4%)	Total (100%)	*p*
Age (years)				
Mean (sd±)	73.7 (±6.3)	74.3 (±5.82)	73.9 (±6.2)	
Median (q_1_, q_3_) ^c^	73.0 (69.0, 78.0)	73 (70.0, 79.0)	73.0 (69, 78)	0.322 ^a^
Marital status				
Married	89 (53.9)	76 (46.1)	165 (45.1)	<0.001 ^b^
Single	74 (93.7)	5 (6.3)	79 (21.6)	
Widow	110 (90.2)	12 (9.8)	122 (33.3)	
Years of schooling				
≤6	89 (82.4)	19 (17.6)	108 (29.5)	0.065 ^b^
7–≤12	96 (73.3)	35 (26.7)	131 (35.8)	
12>	88 (69.3)	39 (30.7)	127 (34.7)	
Smoking				
Yes	19 (61.3)	12 (38.7)	31 (8.5)	<0.001 ^b^
No	185 (88.5)	24 (11.5)	209 (57.1)	
Former smoker	69 (54.8)	57 (45.2)	126 (34.4)	
More than 100 cigarettes smoked				
Yes	66 (54.6)	55 (45.5)	121(33.1)	<0.001 ^b^
No	207 (84.5)	38 (15.5)	245 (66.9)	
Drinking alcoholic beverages				
Yes	12 (40.0)	18 (60.0)	30 (8.2)	<0.001 ^b^
No	261(77.7)	75 (22.3)	336 (91.8)	
Hypertension				
Yes	158 (78.6)	43 (21.4)	201 (54.9)	0.051 ^b^
No	115 (69.7)	50 (30.3)	165 (45.1)	
Diabetes mellitus type 2				
Yes	65 (71.4)	26 (28.6)	91 (24.9)	0.424 ^b^
No	208 (75.6)	67 (24.4)	275 (75.1)	
Nutritional status (MNA)				
Risk of malnutrition/Malnutrition ^d^	124 (83.2)	25 (16.8)	149 (40.71)	0.002 ^b^
Well nourished	149 (68.7)	68 (31.3)	217 (59.3)	
Body mass index (kg/m^2^) Mean (sd±)	27.5 (±4.4)	26.5 (±3.6)	27.3 (±4.2)	
Median (q_1_, q_3_)	27.0 (24.6, 30.1)	26.5 (24.5, 29.0)	26.9 (24.6, 29.9)	0.130 ^a^
Underweight	0	1 (100)	1 (0.27)	0.077 ^b^
Normal	65 (73.9)	23 (26.1)	88 (24.0)	
Overweight	133 (71.5)	53 (28.5)	186 (50.82)	
Obese	75 (82.4)	16 (17.6)	91 (24.86)	
Body fat %				
Mean (sd±)	39.8 (±5.5)	32.2 (±4.0)	37.5 (±7.4)	
Median (q_1_, q_3_)	40 (36.2, 43.3)	32.5 (29.8, 34.8)	37.9 (33.4, 42.5)	<0.001 ^a^
Fat-free mass (lean + BMC) ^e^ (kg) mean (sd±)	36.5 (±3.39)	49.4 (±5.17)	39.8 (±6.85)	
Median (q_1_, q_3_)	36.2 (34.1, 38.5)	49.9 (45.4, 58.2)	49.0 (45.0, 52.8)	<0.001 ^a^
Oral health quality of life (GOHAI)				
Mean (sd±)	38.05 (±8.81)	39.37(±8.61)	39.05 (±8.75)	
Median (q_1_, q_3_)	41 (34.0, 47.0)	42 (33.0, 46.0)	42.0 (34.0, 47.0)	0.772 ^a^
Physical component				
Mean (sd±)	16.4 (±3.8)	17.0 (±3.2)	16.6 (±3.7)	
Median (q_1_, q_3_)	17.0 (14.0, 20.0)	17.0 (15.0, 20.0)	17 (14.0, 20.0)	0.360 ^a^
Psychosocial component				
Mean (sd±)	21.3 (±4.06)	21.2 (±4.45)	21.3 (±4.15)	
Median (q_1_, q_3_)	22.0 (19.0, 25.0)	23 (19.0, 26.0)	22.5 (19.0, 25.0)	0.955 ^a^
Oral pain/discomfort component				
Mean (sd±)	13.2 (±2.21)	13.2 (±1.93)	13.2 (±2.14)	
Median (q_1_, q_3_)	14.0 (11.0, 15.0)	14.0 (12.0, 15.0)	14.0 (12.0, 15.0)	0.650 ^a^
Short physical performance battery				
Mean (sd±)	8.3 (±2.72)	9.2 (±2.49)	8.5 (±2.69)	
Median (q_1_, q_3_)	9.0(7.0, 10.0)	10.0 (8.0, 11.0)	9.0 (7.0, 11.0)	0.005 ^a^
SPPB ≤ 8				
Yes	128 (80.5)	31 (19.5)	159 (43.4)	0.023 ^b^
No	145 (70.0)	62 (30.0)	207 (56.6)	
SPPB subtest				
Chair stand scores				
Mean (sd±)	2.7 (±1.29)	2.9 (±1.23)	2.7 (±1.27)	
Median (q_1_, q_3_)	3 (2, 4)	3 (2, 5)	3 (2, 4)	0.187 ^a^
Balance scores mean (sd±)				
Mean (sd±)	2.6 (±1.38)	3.0 (±1.21)	2.7 (±1.35)	
Median (q_1_, q_3_)	3 (1, 4)	3 (2, 4)	3 (1, 4)	0.011 ^a^
Gait speed scores mean (sd±)				
Mean (sd±)	3.1 (±1.00)	3.2 (±0.95)	3.1 (±0.99)	
Median (q_1_, q_3_)	3 (2, 4)	4 (3, 4)	3 (2, 4)	0.042 ^a^

^a^ Differences in median values between women and men (assessed using *p*-value of Mann–Whitney U-test). ^b^ Differences in categorical variables between women and men (assessed using Chi-2 test). ^c^ (q_1_, q_3_) first quartile, third quartile. ^d^ Six older adults were identified as malnourished. ^e^ BMC: bone mineral content obtained via dual-energy X-ray absorptiometry.

**Table 2:** Characteristics of the participants by physical performance determined by applying short physical performance battery (SPPB).

Characteristics	SPPB > 8	SPPB ≤ 8	*p*
	(207) (56.6%)	(159) (43.4%)	
Age			
Mean (sd±)	72.5 (±5.6)	75.8 (±6.5)	
Median (q_1_, q_3_) ^c^	72 (68.0, 77.0)	76 (70.0, 80.0)	<0.001 ^a^
Sex	n (%)	n (%)	
Women	145 (53.1)	128 (46.9)	0.023 ^b^
Men	62 (66.7)	31(33.3)	
Marital status			
Married	107 (64.9)	58 (35.1)	0.015 ^b^
Single	39 (49.4)	40 (50.6)	
Widow	61(50.0)	61(50.0)	
Years of schooling			
≤6	43 (39.8)	65 (60.2)	<0.001 ^b^
7–≤12	77 (58.8)	54 (41.2)	
12>	87 (68.5)	40 (31.5)	
Tobacco use			
Current smoker	15 (48.4)	16 (51.6)	0.556 ^b^
Non-smoker	122 (58.4)	87 (41.6)	
Former smoker	70 (55.6)	56 (44.4)	
Ever smoked			
≥100 cigarettes	140 (57.1)	105 (42.9)	0.748 ^b^
<100 cigarette	67 (55.4)	54 (44.6)	
Drinking alcoholic beverages			
Yes	20 (66.7)	10 (33.3)	0.244 ^b^
No	187 (55.6)	149 (44.4)	
Hypertension			
Yes	100 (49.7)	101 (50.3)	0.004 ^b^
No	107 (64.9)	58 (35.1)	
Diabetes mellitus type 2			
Yes	40 (44.0)	51 (56.0)	0.005 ^b^
No	167 (60.7)	108 (39.3)	
Mini Nutritional Assessment (MNA)			
Malnutrition/risk of malnutrition	70 (47.0)	79 (53.0)	0.002 ^b^
Normal	137 (63.1)	80 (36.9)	
Body mass index (kg/m^2^)			
Mean (sd±)	26.8 (± 3.9)	27.8 (±4.6)	
Median (q_1_, q_3_)	26.7 (24.3, 29.4)	27.3 (24.8, 30.5)	0.063 ^a^
Underweight	1 (100)	0 (0. 0)	0.167 ^b^
Normal	56 (63.6)	32 (36.4)	
Overweight	106 (57.0)	80 (43.0)	
Obese	44 (48.4)	47 (51.6)	
Body fat %			
Mean (sd±)	32.2 (±4.0)	39.8 (±5.5)	
Median (q_1_, q_3_)	36.3 (32.8, 41.4)	39.5 (35.3, 43.3)	<0.001 ^a^
Fat-free mass (lean + BMC) ^d^ (kg)			
Mean (sd±)	40.4 (±7.1)	39.0 (±6.5)	
Median (q_1_, q_3_)	38.1 (35.4, 44.9)	37.1 (34.3, 41.9)	<0.001 ^a^
Oral health quality of life (GOHAI)			
Mean (sd±)	40.5 (±7.96)	37.1 (±9.36)	
Median (q_1_, q_3_)	43.0 (36.0, 47.0)	39.0 (29.0, 46.0)	0.009 ^a^
Physical component			
Mean (sd±)	17.2 (±3.3)	15.70 (±4.0)	<0.001 ^a^
Median (q_1_, q_3_)	19.0 (15.0, 20.0)	16 (13.0, 20.0)	
Psychosocial component			
Mean (sd±)	21.8 (±4.0)	20.6 (±4.3)	
Median (q_1_, q_3_)	23.0 (20.0, 25.0)	21.0 (18.0, 25.0)	0.007 ^a^
Oral pain/discomfort component			
Mean (sd±)	13.5 (±1.9)	12.8 (±2.4)	
Median (q_1_, q_3_)	14.0 (12.0, 15.0)	13.0(11.0, 15.0)	0.006 ^a^

^a^ For differences in low SPPB score and high SPPB, the Mann–Whitney U-test was applied. ^b^ Differences in categorical variables between low SPPB score and high SPPB score (assessed using Chi-2 test). ^c^ (q_1_, q_3_) first quartile, third quartile. ^d^ BMC: bone mineral content obtained via dual-energy X-ray absorptiometry scan.

**Table 3:** Results of the logistic regression models for physical performance, sociodemographic characteristics, chronic conditions, nutritional status, and perceived oral health-related quality of life.

	Bivariate Models ^a^			Multiple Model ^c^		
Characteristics	OR ^b^	(95%CI)	*p*	OR ^d^	(95%CI)	*p*
Age	1.10	(1.06–1.14)	<0.001	1.13	(0.70–2.22)	<0.001
Sex						
Men	1			1		
Women	1.7	(1.08–2.89)	0.024	1.13	(1.08–1.18)	0.732
Tobacco consumption						
Smoking < 100 cigarettes	1			1		
Smoking ≥ 100 cigarettes	1.37	(0.84–2.24)	0.206	1.29	(0.77–2.18)	0.333
Alcohol consumption						
No	1			1		
Yes	0.72	(0.31–1.68)	0.440	1.01	(0.41–2.43)	0.991
Hypertension						
No	1			1		
Yes	1.59	(1.02–2.48)	0.041	1.09	(0.66–1.78)	0.740
Type 2 diabetes mellitus						
No	1			1		
Yes	2.45	(1.46–4.11)	0.001	2.10	(1.20–3.67)	0.009
Nutritional status normal MNA ≥ 24 ^e^	1			1		
Risk of malnutrition/malnutrition MNA ≤ 23.5	1.92	(1.22 -3.01)	0.005	1.76	(1.01–3.09)	0.047
DXA body fat% ^f^	1.08	(1.03–1.13)	0.001	1.09	(1.04–1.14)	<0.001
Oral health-related quality of life						
GOHAI ≥ 37 ^g^	1			1		
GOHAI < 37	2.18	(1.35–3.51)	0.001	1.96	(1.18–3.24)	0.009

^a^ Bivariate model with short physical performance as dependent variable (cut-off value SPPB ≤ 8). ^b^ Crude odds ratios. ^c^ Multivariate model. Goodness-of-fit Hosmer–Lemeshow test *p* = 0.786 ^d^ Odds ratios adjusted for age, sex, alcohol and tobacco consumption, hypertension, type 2 diabetes, body fat percentage, and oral health-related quality of life score. Mean Variance Inflation Factor, VIF =1.21 ^e^ MNA: Mini Nutritional Assessment. ^f^ Body fat percentage as determined via dual-energy X-ray absorptiometry. ^g^ GOHAI: General Oral Health Assessment Instrument.

## Data Availability

The data are available from the corresponding authors M.E.I.C. and M.C.-A., upon reasonable requests.
